# AGETECH AND THE ETHICAL IMPERATIVE

**DOI:** 10.1093/geroni/igad104.1035

**Published:** 2023-12-21

**Authors:** Charlene Chu, Jennifer Boger, Andrew Sixsmith

## Abstract

“AgeTech” refers to technological trends in e-health, robotics, artificial intelligence (AI), and mobile technologies” as a way to improve the health, well-being, independence, and social participation of older people. This symposium provides an overview of the imperative to integrate ethics into the design and development of technology, from start to finish. The first talk presents reflections from older adult members of two AgeTech groups: AGE-WELL’s Older Adults and Caregivers Advisory Committee, and the Research Group of Seniors 411, which will outline the ethical issues and ageism experienced by older adults. To further examine these issues, the second presentation explores a framework for how ageist attitudes can be encoded and perpetuated as digital ageism, both in AI and other technologies, and highlights a need for broader community inclusion. The third paper explores the challenges of virtual community inclusion, highlighting the challenges and benefits of digital participation in a post-COVID world. A health equity focus will be applied when highlighting the unique needs of seldom-heard and under-served older people in virtual spaces to enhance social inclusion. The last paper will highlight the “ethical by design” approach that will encourage and support culture change within AgeTech research and industry. This symposium will contribute to a more ethical, and inclusive design of technologies to support healthy aging.

